# Biomimetic oxygen delivery nanoparticles for enhancing photodynamic therapy in triple-negative breast cancer

**DOI:** 10.1186/s12951-021-00827-2

**Published:** 2021-03-20

**Authors:** Hanyi Fang, Yongkang Gai, Sheng Wang, Qingyao Liu, Xiao Zhang, Min Ye, Jianling Tan, Yu Long, Kuanyin Wang, Yongxue Zhang, Xiaoli Lan

**Affiliations:** 1 Department of Nuclear Medicine, Union Hospital, Tongji Medical College, Huazhong University of Science and Technology, Wuhan, 430022 China; 2 Hubei Province Key Laboratory of Molecular Imaging, Wuhan, 430022 China; 3 School of Pharmacy, Tongji Medical College, Huazhong University of Science and Technology, Wuhan, 430030 China

**Keywords:** Triple-negative breast cancer, Cancer cell membranes, Photodynamic therapy, Hypoxia, Oxygen delivery, Nanoprobes

## Abstract

**Background:**

Triple-negative breast cancer (TNBC) is a kind of aggressive breast cancer with a high rate of metastasis, poor overall survival time, and a low response to targeted therapies. To improve the therapeutic efficacy and overcome the drug resistance of TNBC treatments, here we developed the cancer cell membrane-coated oxygen delivery nanoprobe, CCm–HSA–ICG–PFTBA, which can improve the hypoxia at tumor sites and enhance the therapeutic efficacy of the photodynamic therapy (PDT), resulting in relieving the tumor growth in TNBC xenografts.

**Results:**

The size of the CCm–HSA–ICG–PFTBA was 131.3 ± 1.08 nm. The in vitro ^1^O_2_ and ROS concentrations of the CCm–HSA–ICG–PFTBA group were both significantly higher than those of the other groups (*P* < 0.001). In vivo fluorescence imaging revealed that the best time window was at 24 h post-injection of the CCm–HSA–ICG–PFTBA. Both in vivo ^18^F-FMISO PET imaging and ex vivo immunofluorescence staining results exhibited that the tumor hypoxia was significantly improved at 24 h post-injection of the CCm–HSA–ICG–PFTBA. For in vivo PDT treatment, the tumor volume and weight of the CCm–HSA–ICG–PFTBA with NIR group were both the smallest among all the groups and significantly decreased compared to the untreated group (*P* < 0.01). No obvious biotoxicity was observed by the injection of CCm–HSA–ICG–PFTBA till 14 days.

**Conclusions:**

By using the high oxygen solubility of perfluorocarbon (PFC) and the homologous targeting ability of cancer cell membranes, CCm–HSA–ICG–PFTBA can target tumor tissues, mitigate the hypoxia of the tumor microenvironment, and enhance the PDT efficacy in TNBC xenografts. Furthermore, the HSA, ICG, and PFC are all FDA-approved materials, which render the nanoparticles highly biocompatible and enhance the potential for clinical translation in the treatment of TNBC patients.

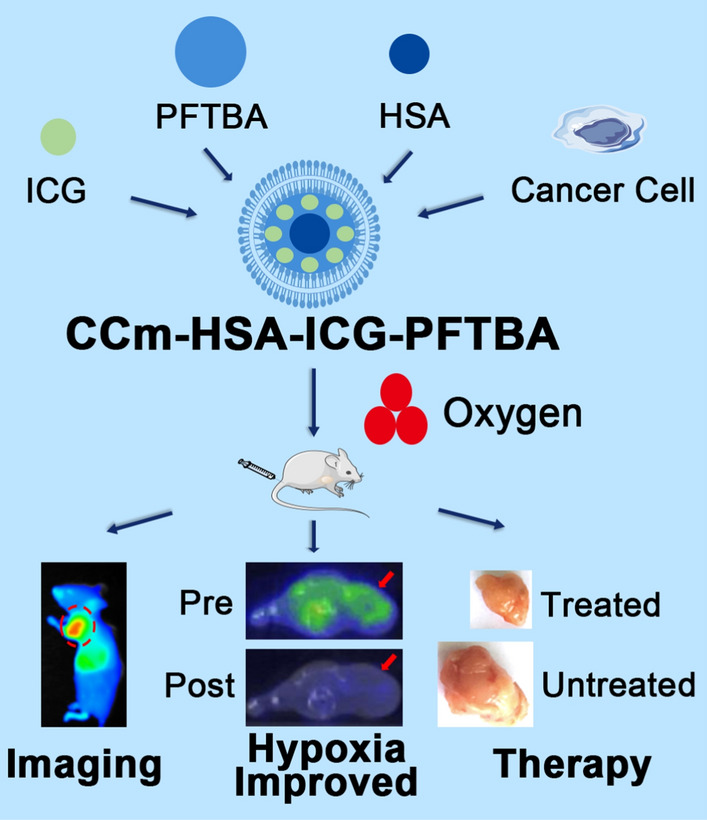

**Supplementary Information:**

The online version contains supplementary material available at 10.1186/s12951-021-00827-2.

## Background

Breast cancer is the most common malignant disease in women, which caused the second most death cases of tumors in women worldwide in 2020 [1]. Triple-negative breast cancer (TNBC) is a subtype of breast cancer and accounts for 15% of all breast cancer patients [2]. Due to the absence of expressions of the estrogen receptor (ER), progesterone receptor (PR), and human epidermal growth factor receptor 2 (HER2), TNBC patients have a high rate of metastasis and recurrence, poor overall survival time, and are lack of chances for targeted therapy [3]. Despite conventional therapies of breast cancer patients, including the chemotherapy, endocrine therapy, and targeted therapy, photodynamic therapy (PDT) is a new choice to improve the therapeutic efficacy and overcome the drug resistance to the TNBC treatment [4].

PDT is a rapidly developing and clinically approved cancer treatment [5] where the anti-tumor effects of the PDT depend on the reactive oxygen species (ROS) and the singlet oxygen (^1^O_2_) generated by the oxygen in the photodynamic reaction to enhance cell killing during PDT [6, 7]. However, PDT does consume oxygen and initiates vascular shutdown, which translates to less oxygen and worsening hypoxia [8, 9]. Therefore, the development of effective strategies to overcome a hypoxic tumor microenvironment is highly sought after to achieve excellent anti-tumor therapy efficacy.

Perfluorocarbons (PFC), with the ability to extend the half-life time of the ^1^O_2_ to approximately 10^5^-fold to other solvents [10], is an ideal carrier for oxygen delivery [11]. Further, PFC is a highly biocompatible and inert chemical compound and has been widely used in the clinic for the contrast-enhanced ultrasound imaging and prevention of ischemia/reperfusion in tissue and organ injuries [12]. PFC is supposed to be helpful to enhance the efficacy of PDT by delivering oxygen.

A proper photosensitizer is required to perform PDT. For deep tissue penetration and low autofluorescence, near-infrared (NIR) light (700–900 nm) is usually preferred as the excitation wavelength range [13]. Indocyanine green (ICG), a kind of NIR photosensitizer, is the U.S. Food and Drug Administration (FDA)-approved dye for blood volume measurement [14]. Nevertheless, fluorescence quenching often occurs because of ICG aggregation and short-time blood circulation [15]. To mitigate this obstacle, human serum albumin (HSA) has been employed to increases the stability of ICG and prolongs circulation time [16, 17]. In addition to PFC, ICG, and HSA are all FDA-approved for human use, thereby facilitating the usage of these materials clinically.

**Scheme 1.**
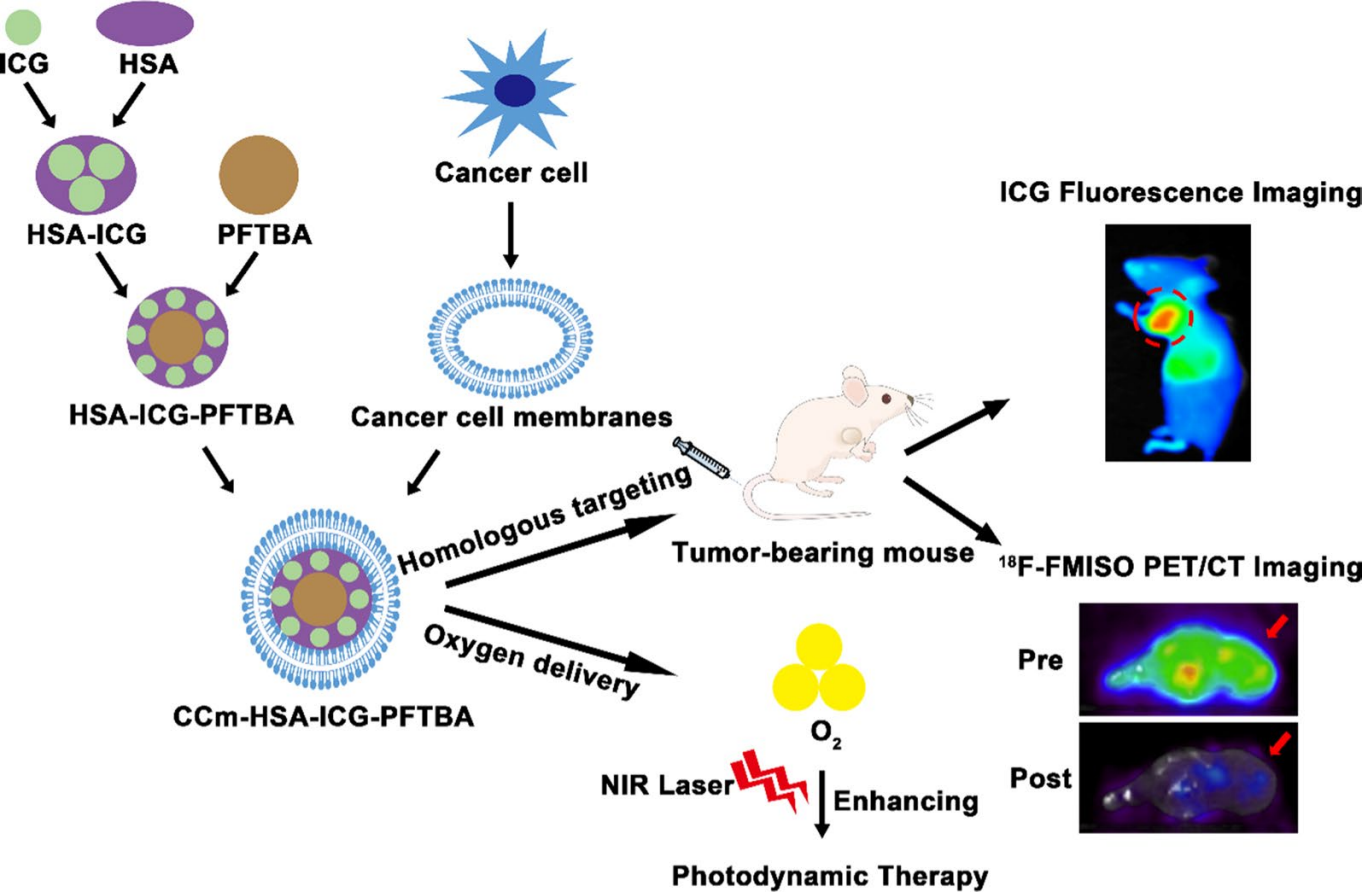
Illustration of the biomimetic oxygen-delivery nanoprobe. It was cancer cell membrane-coated indocyanine green-doped perfluorocarbon (CCm–HSA–ICG–PFTBA) for homologous targeting and improving oxygen concentration at tumor sites. ^18^F-FMISO PET/CT imaging was performed to measure the hypoxia in vivo. CCm–HSA–ICG–PFTBA was injected into 4T1 xenografts and then photodynamic therapy was performed. Tumor volume was measured to evaluate the therapeutic efficacy enhancement

Immune evasion and specific tumor-targeting characteristics remain challenges to tackle to maximize the efficacy of PDT. Our previous work revealed that cancer cell membranes (CCm) might possess the desired immune evasion and homologous targeting characteristics [18, 19]. Here, we designed the biomimetic oxygen delivery nanoprobe, namely cancer cell membrane-coated HSA–ICG–doped perfluorotributylamine (CCm–HSA–ICG–PFTBA) for homologous targeting and hypoxia relieving at tumor sites. A non-invasive and dynamic ^18^F-fluoromisonidazole (^18^F-FMISO) positron emission tomography/computed tomography (PET/CT) imaging was performed to measure hypoxia levels at tumor sites in vivo [20, 21]. We concurrently used CCm–HSA–ICG–PFTBA for PDT in 4T1 mice xenografts to observe the enhanced therapeutic efficacy because of the relieved oxygenation at the tumor sites (Scheme 1).

## Results

### Preparation and characterization of CCm–HSA–ICG–PFTBA

**Fig. 1:**
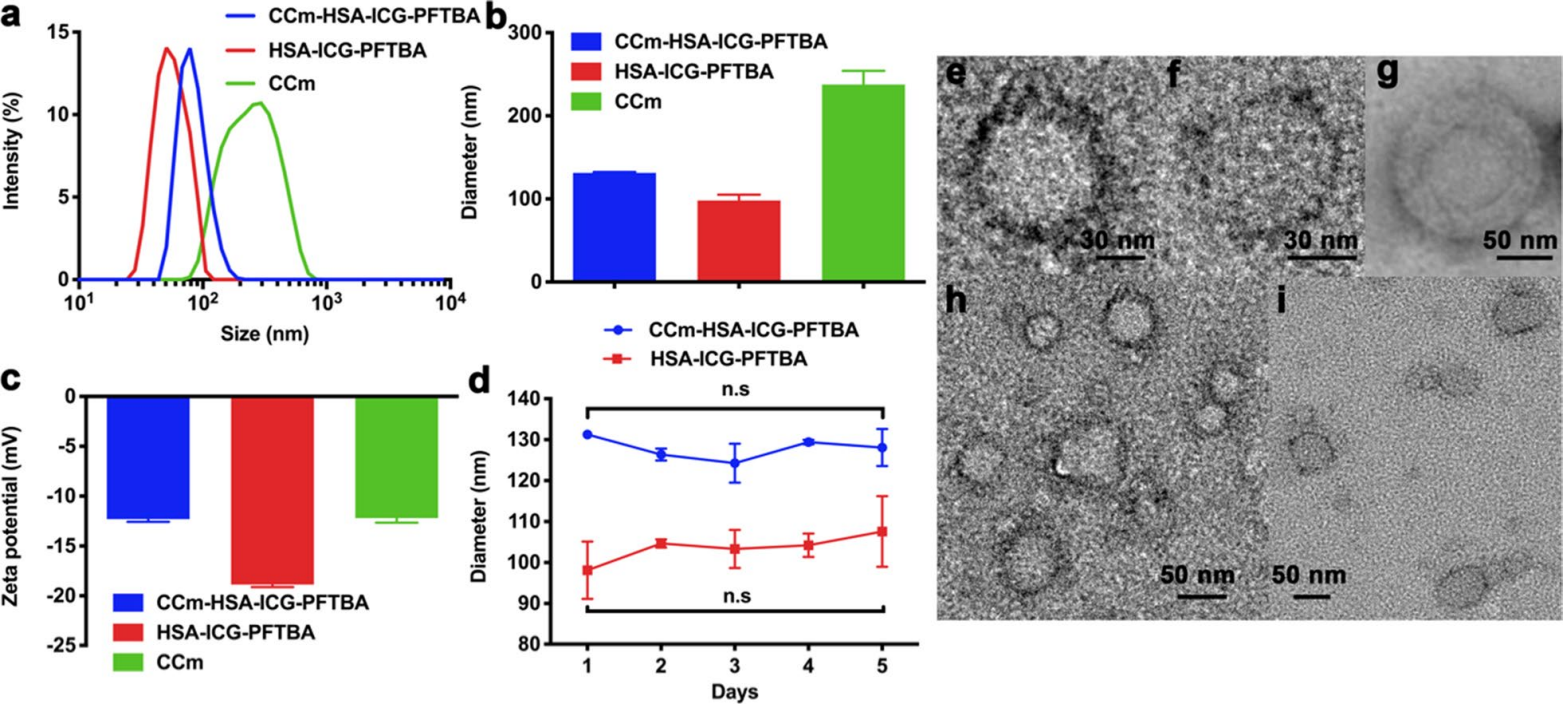
Characterization of CCm–HSA–ICG–PFTBA. **a** Size intensity curves, **b** hydrodynamic size, **c** Zeta potential of CCm–HSA–ICG–PFTBA, HSA–ICG–PFTBA, and cancer cell membrane (CCm). **d** Stability of CCm–HSA–ICG–PFTBA and HSA–ICG–PFTBA. **e**–**i** TEM images of **e**–**h** CCm–HSA–ICG–PFTBA, **f**–**i** HSA–ICG–PFTBA, and **g** CCm. Scale bars = 30 nm and 50 nm. Data are represented as mean ± SD (n = 3)

HSA was used as a carrier to stabilize ICG and PFTBA. The HSA–ICG–PFTBA was prepared by stirring and ultrasonic. CCm were processed from 4T1 cells using a procedure described in our previous study [18]. CCm–HSA–ICG–PFTBA was produced via physical extrusion [18]. Dynamic light scattering (DLS) showed that the hydrodynamic size of HSA–ICG–PFTBA was 98.11 ± 6.99 nm, while that of CCm–HSA–ICG–PFTBA was 131.3 ± 1.08 nm (Fig. 1a, b). The zeta potential results revealed that the surface potential of CCm–HSA–ICG–PFTBA was similar to that of the CCm (Fig. 1c), indicating that CCm had been successfully coated onto the surface of the HSA–ICG–PFTBA. Both CCm–HSA–ICG–PFTBA and HSA–ICG–PFTBA exhibited good stability of hydrodynamic size stored in the phosphate-buffered saline (PBS) for 5 days (Fig. 1d). The structures of both CCm–HSA–ICG–PFTBA and HSA–ICG–PFTBA were verified by the transmission electron microscopy (TEM) (Fig. 1e–i).

**Fig. 2:**
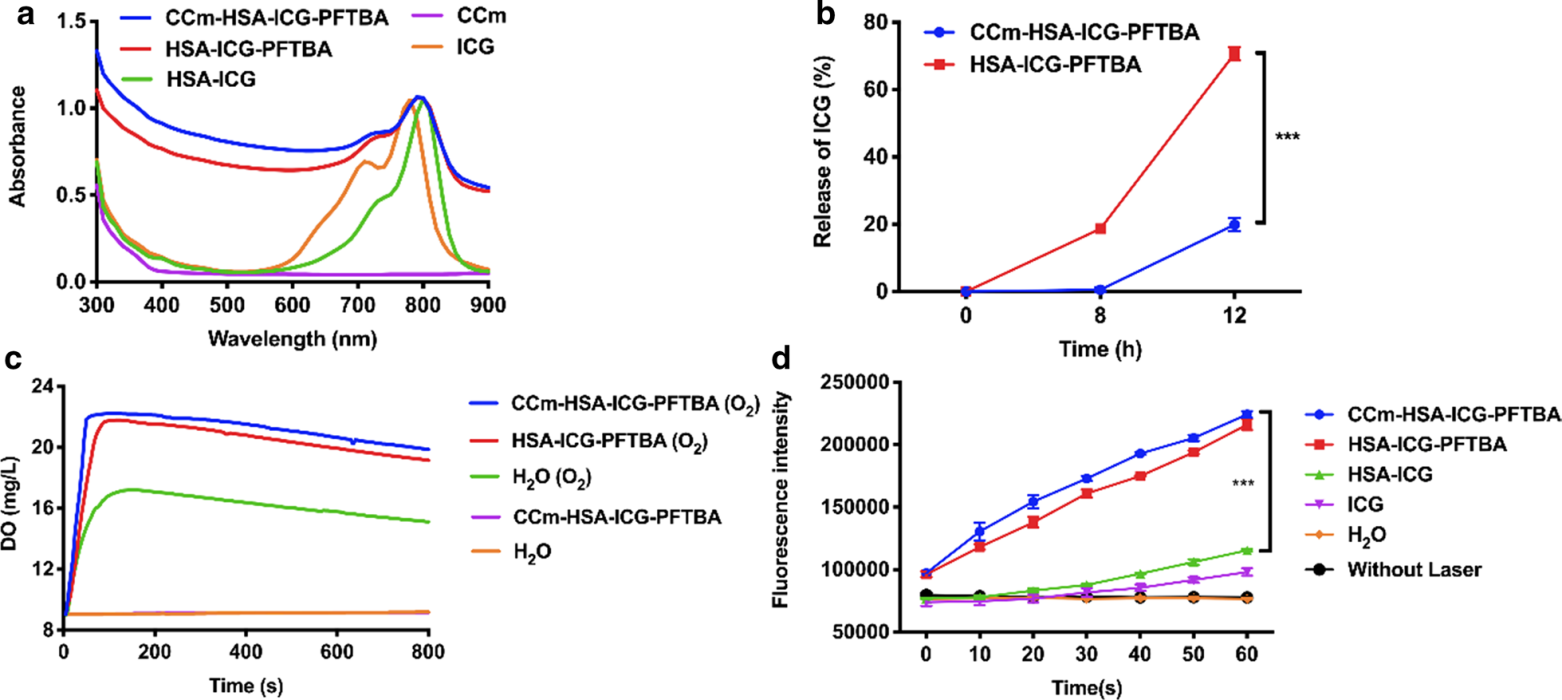
Properties of CCm–HSA–ICG–PFTBA. **a** UV–vis absorbance spectra of CCm–HSA–ICG–PFTBA, HSA–ICG–PFTBA, HSA–ICG, CCm, and ICG. **b** The release of CCm–HSA–ICG–PFTBA and HSA–ICG–PFTBA in serum under 37 ℃. **c** Oxygen concentrations in water after adding CCm–HSA–ICG–PFTBA and HSA–ICG–PFTBA with or without pre-oxygenation. **d** Enhanced ^1^O_2_ generation of CCm–HSA–ICG–PFTBA, HSA–ICG–PFTBA, HSA–ICG, and ICG. Data are represented as mean ± SD (n = 3). *** indicates *P* < 0.001

The characteristic peak of the ICG was observed in the CCm–HSA–ICG–PFTBA by UV–vis and fluorescence spectra (Fig. 2a), suggesting that CCm coating had no impact on the optical properties of the ICG. In dark conditions, the ICG peaks of the CCm–HSA–ICG–PFTBA, HSA–ICG–PFTBA, and HSA–ICG showed nearly no degradation, while 63% degradation was observed in the ICG water solution (Additional file 1: Fig. S1a–e), demonstrating that ICG degradation was overcame because of the help of HSA. The release study was conducted in the serum at 37 ℃, where approximately 70% of ICG was released from the HSA–ICG–PFTBA after 12 h of incubation, which was significantly higher than that of the CCm–HSA–ICG–PFTBA (approximately 20%, *P* < 0.001, Fig. 2b), indicating that cancer cell membrane coating conferred stability and lowed ICG leakage of this nanoprobe. To verify the oxygen enhancement ability, the oxygen concentrations in different solutions were measured. Higher oxygen concentration and faster oxygen increasing rate were observed in the preoxygenated CCm–HSA–ICG–PFTBA added group (from 9 to 22.23 mg/L within 100 s) than that of the same amount of the preoxygenated water added group (from 9 to 17.23 mg/L within 150 s) (Fig. 2c). These results showed that CCm–HSA–ICG–PFTBA exhibited the ability to enhance oxygen concentration.

### In vitro ^1^O_2_ and ROS evaluation

**Fig. 3:**
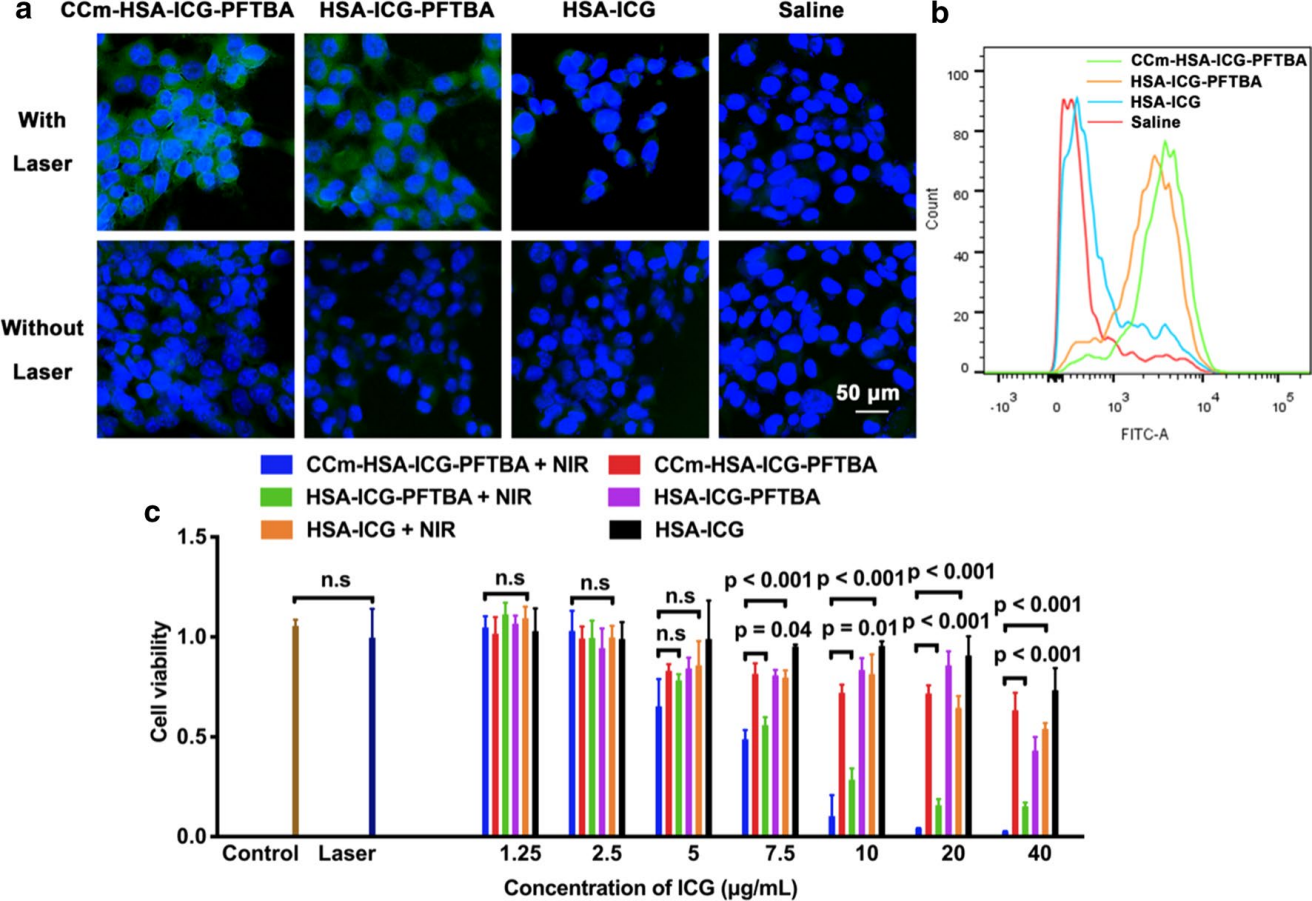
In vitro experiments of CCm–HSA–ICG–PFTBA. **a** The confocal laser scanning microscope images of ROS generation in cells treated with the CCm–HSA–ICG–PFTBA, HSA–ICG–PFTBA, HSA–ICG, and saline with or without NIR laser irradiation. Green fluorescence represented DCFH-DA, showing ROS concentrations, and blue fluorescence represented DAPI, showing cell nucleus. The scale bar = 50 μm. **b** Flow cytometry analysis of ROS generation in cells treated with the CCm–HSA–ICG–PFTBA, HSA–ICG–PFTBA, HSA–ICG, and saline with NIR laser irradiation. **c** Cell viability after treated with the CCm–HSA–ICG–PFTBA, HSA–ICG–PFTBA, and HSA–ICG with or without NIR laser irradiation. Data are represented as mean ± SD (n = 5)

To measure the ^1^O_2_ generation ability of the CCm–HSA–ICG–PFTBA, an ^1^O_2_ indicator, Singlet Oxygen Sensor Green (SOSG) was used. By using the NIR laser irradiation, the fluorescence of the CCm–HSA–ICG–PFTBA and HSA–ICG–PFTBA significantly increased than that of the HSA–ICG (*P* < 0.001, Fig. 2d), demonstrating the higher ^1^O_2_ generation capability resulted from the PFTBA. To measure the ROS concentration, we used the dichloro-dihydro-fluorescein diacetate (DCFH-DA) as an indicator. 4T1 cells were incubated with DCFH-DA, which is prone to cleavage by intracellular esterases into 2,7-dichlorofluorescin (H_2_DCF) [22]. After the NIR laser irradiation, the generated ROS oxidized H_2_DCF to DCF, producing green fluorescence, of an intensity that was directly proportional to the ROS concentration [22]. As shown in Fig. 3a, with NIR laser irradiation, strong green fluorescence was observed in the CCm–HSA–ICG–PFTBA and HSA–ICG–PFTBA groups, while that of HSA–ICG and saline groups were negligible. These results coincided with those from the flow cytometry, where ample higher fluorescence intensity was observed in the CCm–HSA–ICG–PFTBA (94.5%) and HSA–ICG–PFTBA (89.0%) groups, compared with that of the HSA–ICG (28.3%) and saline control (20%, Fig. 3b). The results of the high ^1^O_2_ and ROS concentration indicated that CCm–HSA–ICG–PFTBA could enhance the PDT efficacy in vitro by the high oxygen capacity of PFTBA.

### In vitro cytotoxicity

To evaluate the cytotoxicity of PDT enhancement, 4T1 cells were incubated with samples at different concentrations with or without NIR irradiation, and cell viabilities were measured by a cell counting kit-8 (CCK-8). As shown in Fig. 3c, all concentrations without NIR irradiation exhibited negligible toxicity to the cells. For the groups received NIR irradiation, the toxicity of CCm–HSA–ICG–PFTBA was significantly higher than that of the HSA–ICG–PFTBA and HSA–ICG when the concentration of ICG was higher than 7.5 μg/mL. And the higher the concentration was, the significantly higher the toxicity was shown.

### In vivo fluorescence imaging

**Fig. 4:**
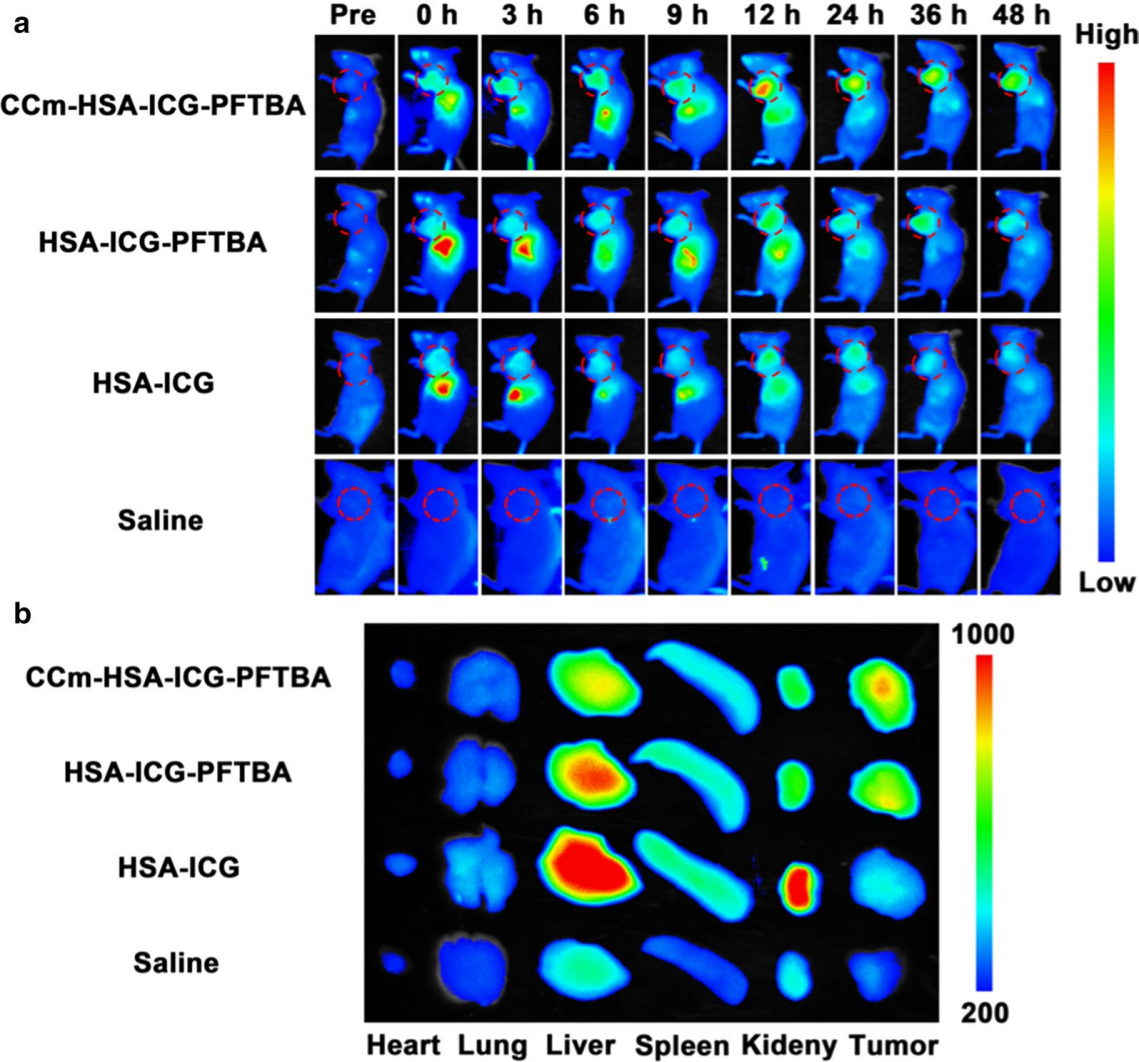
Fluorescence imaging. **a** In vivo fluorescence images of TNBC xenografts after the injection of the CCm–HSA–ICG–PFTBA, HSA–ICG–PFTBA, HSA–ICG, and saline at different time points. Red circles indicated the tumor site. **b** Ex vivo fluorescence images of major organs and tumors at 48 h post-injection

To determine the best time window for PDT, we examined the in vivo tumor distribution of CCm–HSA–ICG–PFTBA in 4T1 mice xenografts. The ICG fluorescence signals were obtained at 0, 3, 6, 9, 12, 24, 36, and 48 h post-injection of CCm–HSA–ICG–PFTBA, HSA–ICG–PFTBA, HSA–ICG, and saline, all via tail veins. We found that the tumor fluorescence of the CCm–HSA–ICG–PFTBA group was stronger than that of other groups and lasted till 48 h post-injection (Fig. 4a). The tumor fluorescence of the HSA–ICG group rapidly faded away and the residual signal in the tumor site was ascribed to blood pool emissions. The liver is well-known as one of the primary sites of the phagocyte-enriched reticuloendothelial system (RES) [23] and hence can accumulate most of the exogenous materials [24]. Liver accumulation of the CCm–HSA–ICG–PFTBA was much lower than that of the HSA–ICG–PFTBA, indicating that cancer cell membrane coating decreased the RES uptake. At 48 h post-injection, the main organs and tumors were collected for the ex vivo fluorescence imaging. As shown in Fig. 4b, the tumor fluorescence was higher and the liver and spleen fluorescence was lower in the CCm–HSA–ICG–PFTBA group compared with other groups. The fluorescence imaging results indicated that CCm–HSA–ICG–PFTBA could homologously target tumors and enhance the immune evasion.

### In vivo and ex vivo tumor oxygenation enhancement

**Fig. 5:**
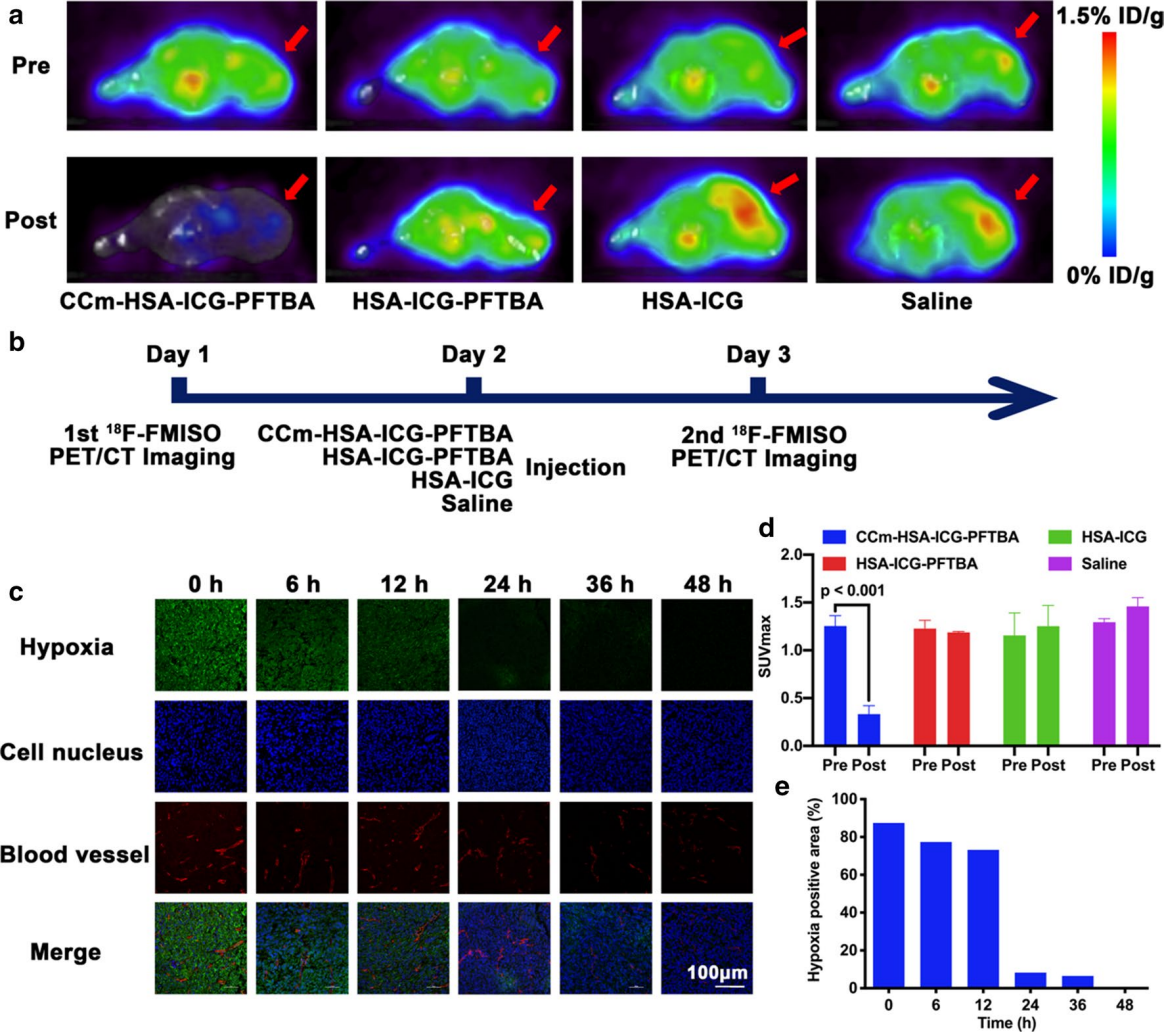
Hypoxia improvement at tumor sites. **a** In vivo transverse ^18^F-FMISO PET/CT images of TNBC xenografts before and after 24 h injection of the CCm–HSA–ICG–PFTBA, HSA–ICG–PFTBA, HSA–ICG, and saline. Red arrows indicated tumor sites. **b** Scheme of the PET/CT imaging. **c** Immunofluorescence images of tumor slices stained by the hypoxyprobe. The blood vessels and hypoxia areas were stained with anti-CD31 antibody (red) and antipimonidazole antibody (green), respectively. Scale bar = 150 μm. **d** The quantitative analysis of SUVmax at tumor sites of CCm–HSA–ICG–PFTBA, HSA–ICG–PFTBA, HSA–ICG, and saline groups in the pre and post ^18^F-FMISO PET/CT imaging. **e** Quantification of tumor hypoxia densities for different time points

After verifying the in vivo distribution of these nanoprobes, we aimed to confirm whether the hypoxia of the tumor microenvironment was relieved. ^18^F-FMISO PET imaging is widely used for measuring in vivo tumor hypoxia [25, 26]. Tumor hypoxia is heterogeneous and exhibits complex dynamic changes during tumor growth [27]. For ^18^F-FMISO PET/CT imaging, more uptake corresponds to higher hypoxia level. The ^18^F-FMISO PET/CT imaging prior to the injection of nanoprobes showed high tumor radioactivity uptake in all groups (Fig. 5a). Then, CCm–HSA–ICG–PFTBA, HSA–ICG–PFTBA, HSA–ICG, and saline were administered to the mice via tail veins. At 24 h post-injection, ^18^F-FMISO PET/CT imaging was performed for a second time (Fig. 5b). A global decrease of radioactivity across the whole body, including the tumor site, was shown after the injection of the CCm–HSA–ICG–PFTBA. The tumor uptake showed no obvious changes after the injection of the HSA–ICG–PFTBA, while there was a slight increase tumor uptake after the injection of HSA–ICG and saline, which due to the fast tumor growth of 4T1 xenografts (Fig. 5a). Considering the fast tumor growth and the imaging results of the HSA–ICG and saline groups, the tumor hypoxia was slightly relieved after the injection of the HSA–ICG–PFTBA. After drawing the ROIs and quantitatively analyzing the SUVmax at the tumor sites, the post SUVmax of tumor (0.33 ± 0.09) was significantly lower than that of the pre SUVmax of tumor (1.25 ± 0.11) in the CCm–HSA–ICG–PFTBA group (*P* < 0.001, Fig. 5d). The liver radioactivity uptake was also significantly reduced after the injection of CCm–HSA–ICG–PFTBA at 24 h post-injection (*P* < 0.001, Additional file 1: Fig. S2a, b). There were no significant differences between the pre and post SUVmax of tumor and liver in other groups (Fig. 5d and Additional file 1: Fig. S2b).

Ex vivo tumor slices of each group were obtained to further confirm the oxygen concentration by using a hypoxia-probe (pimonidazole hydrochloride) for immunofluorescence staining. The hypoxia areas showed obvious reduction, from 87.4% before injection to 8.3% 24 h post-injection of the CCm–HSA–ICG–PFTBA (Fig. 5c, e), with a tenfold decrease. Less improvement of hypoxia was observed in the HSA–ICG–PFTBA and HSA–ICG group (Additional file 1: Fig. S3). It is noticed that the fluorescence of hypoxia areas (green) and blood vessels (red) both decreased, which was due to the vascular shutdown effects during the PDT [8]. These above results indicated that CCm–HSA–ICG–PFTBA could relief tumor hypoxia and therefore could be an ideal strategy to enhance PDT efficacy.

### In vivo PDT efficacy evaluation

**Fig. 6:**
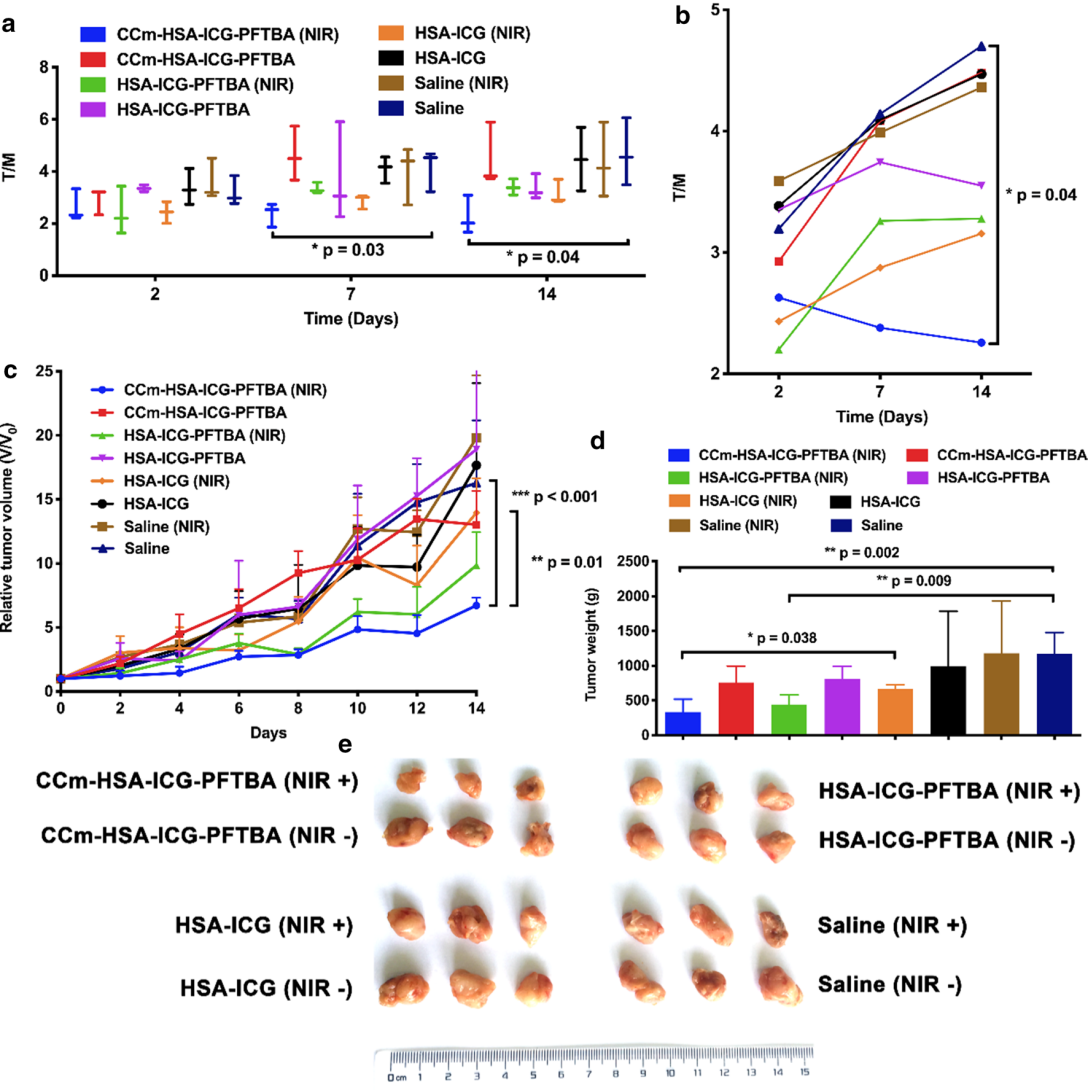
In vivo PDT of tumor-bearing mice. **a**, **b** The tumor to muscle (T/M) ratios of mice after treated with CCm–HSA–ICG–PFTBA, HSA–ICG–PFTBA, HSA–ICG, and saline and with or without NIR laser irradiation. T/M was calculated by drawn ROI in the images of ^18^F-FDG PET images. Values are the means ± SD (n = 3). **c** Relative tumor volume, **d** the tumor weight obtained after treatment for 14 days, and **e** representative photographs of tumor tissues of the mice in different treatment groups. Data are represented as mean ± SD (n = 6). *, **, and *** indicate *P* < 0.05, *P* < 0.01 and *P* < 0.001, respectively

The in vivo PDT efficacy evaluation was performed on 4T1 xenografts. The mice were randomly divided into eight groups, and injected with CCm–HSA–ICG–PFTBA, HSA–ICG–PFTBA, HSA–ICG, and saline (Day 0), respectively, with or without NIR laser irradiation at 24 h post-injection (Day 1). ^18^F-FDG PET imaging was performed to monitor tumor burden in Day 2, Day 7, and Day 14 (Additional file 1: Fig. S4). On Day 7 and Day 14, the tumor-to-muscle radioactivity (T/M) ratio of the CCm–HSA–ICG–PFTBA with NIR group was significantly lower than that of the saline group (*P* = 0.03 and *P* = 0.04 on Day 7 and 14, respectively, Fig. 6a), and gradually decreased, while that of the other groups all increased (Fig. 6b). The tumor volumes were normalized to their initial size. As shown in Fig. 6c, the normalized tumor volumes of the CCm–HSA–ICG–PFTBA with NIR group showed significantly slower increase (*P* = 0.01) than that of the HSA–ICG with NIR group and saline control (*P* < 0.001) on Day 14 (Fig. 6c). There were no significant differences between the CCm–HSA–ICG–PFTBA and HSA–ICG–PFTBA without NIR groups, and the HSA–ICG and saline with or without NIR groups. On Day 14, all the mice were sacrificed and the tumors were weighed. The mean tumor weight of the CCm–HSA–ICG–PFTBA with NIR group was significantly lower than that of the HSA–ICG with NIR group and saline control (*P* = 0.038 and *P* = 0.002, respectively; Fig. 6d). There were no significant differences between other groups. The photos of the tumors were shown in Fig. 6e. These results suggested that CCm–HSA–ICG–PFTBA could enhance the PDT efficacy and relief the tumor growth.

**Fig. 7:**
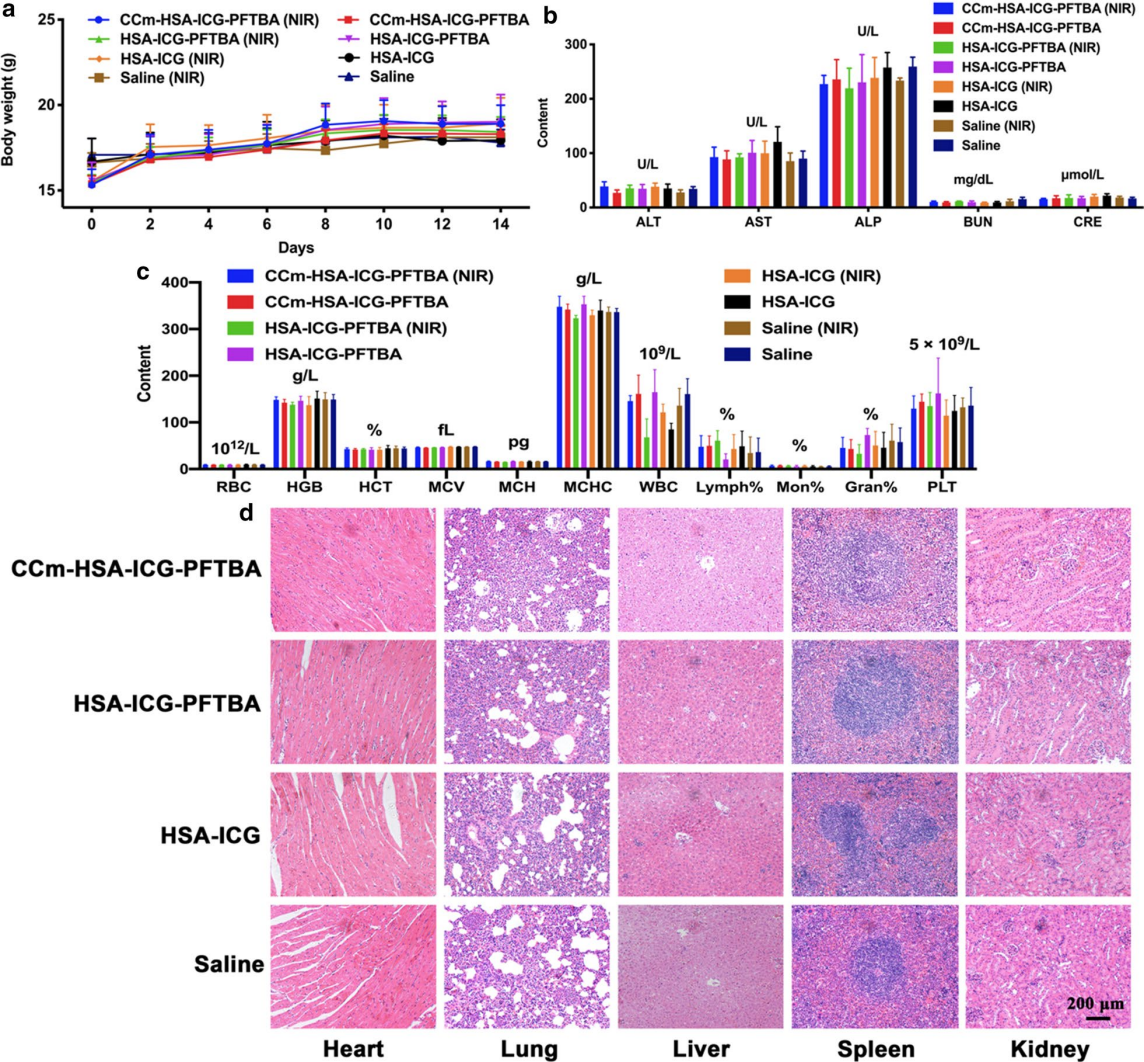
In vivo toxicity evaluation. **a** Mice body-weight-change curves over 14 days after injection with CCm–HSA–ICG–PFTBA, HSA–ICG–PFTBA, HSA–ICG, and saline, with or without NIR irradiation. **b** Blood parameters data. Red blood cells (RBC), hemoglobin (HGB), hematocrit (HCT), mean corpuscular volume (MCV), mean corpuscular hemoglobin (MCH), and mean corpuscular hemoglobin concentration (MCHC). **c** Blood biochemistry data. Alanine transaminase (ALT), aspartate aminotransferase (AST), alkaline phosphatase (ALP), blood urea nitrogen (BUN), and creatinine (CRE). **d** White blood cells (WBC), lymphocytes percentage (Lymph%), monocyte percentage (Mon%), and neutrophil percentage (Neu%). **e** Platelets (PLT). **f** H&E-stained slice images of major organs. Scale bars = 200 μm. Data are represented as mean ± SD (n = 6)

Neither death nor significant decrease in body weight was observed in all groups during the 14 days duration of the experiment (Fig. 7a). On Day 14, all the mice were euthanized and their blood and major organs were collected for blood tests and hematoxylin and eosin (H&E) staining. There was no significant difference between the treatment and the saline control groups in blood parameters and blood chemistry indicators (Fig. 7b–e). Furthermore, no noticeable organ damage was observed on H&E-stained slices (Fig. 7f). Hence, these results showed no obvious toxicity of CCm–HSA–ICG–PFTBA in vivo.

## Discussion

In this study, we designed a cancer cell membrane-coated oxygen delivery nanoprobe CCm–HSA–ICG–PFTBA, which exhibited appropriate structural characteristics, stable optical properties, high biocompatibility, and showed no obvious biotoxicity, making it ideal for biomedical applications. With the homologous targeting and immune evasion abilities from the cancer cell membrane coating, and the oxygen delivery function of the PFTBA, the CCm–HSA–ICG–PFTBA improved the hypoxia in the tumor environment and enhanced the therapeutic efficacy of PDT in TNBC xenografts, indicating that CCm–HSA–ICG–PFTBA was able to contribute to the development of TNBC treatment.

In addition, coating with cell membrane can also stabilize the nanoprobes. In this study, the release of ICG in the bare HSA–ICG–PFTBA was 70%, which was 3.5-fold than that of the CCm–HSA–ICG–PFTBA (the release was 20%) at 12 h after dialysis in the serum. Wu et al*.* also reported that cancer cell membrane coating can suppress the release of doxorubicin and icotinib loaded into the nanoparticles [28]. The slow release of drug encapsulated in the nanoparticles can ensure the long circulation in the bloodstream and reduce systemic toxicity, which makes biomimetic nanoparticles become a powerful vehicle for drug delivery in cancer treatment.

^18^F-FMISO PET/CT imaging was performed to measure the hypoxia at tumor sites in vivo. The radioactivity decreased throughout the whole body including the tumor and liver after injection of CCm–HSA–ICG–PFTBA. This can be explained that, with cancer cell membrane coating, CCm–HSA–ICG–PFTBA was more stable, and the blood circulation time was prolonged, and then some of them can also be captured by RES and circulated in the bloodstream throughout the whole body, which leading to increase the overall oxygenation levels. Although to some extent the cancer cell membrane coating can reduce the RES uptake, the excretion pathway of CCm–HSA–ICG–PFTBA is mostly based on the liver. Due to the oxygen delivery ability of PFTBA, the liver oxygenation level increased, resulting in the highly decreased liver radioactivity uptake of ^18^F-FMISO.

The tumor radioactivity uptake showed no obvious changes after the injection of HSA–ICG–PFTBA. According to the fast tumor growth and the imaging results of the HSA–ICG and saline groups, the tumor hypoxia can be considered slightly relieved after the injection of the HSA–ICG–PFTBA. It can be explained that the HSA–ICG–PFTBA cannot uniformly distribute throughout the whole body due to the instability of the nanoprobe and immune system clearance because of the absence of cancer cell membrane coating, which was in accordance with the results of the ICG release study (Fig. 2b). The slightly improvement of tumor hypoxia after the injection of the HSA–ICG–PFTBA can be attributed to the enhanced permeability and retention (EPR) effect of solid tumors, which in any case was still less effective than the homologous targeting of the CCm–HSA–ICG–PFTBA. It is noticed that for the immunofluorescence staining, the fluorescence of hypoxia areas (green) and blood vessels (red) both decreased, which was due to the vascular shutdown effects during the PDT. The ROS generated during PDT can damage vascular endothelial cells and cause the vascular shutdown, which is an important PDT mechanism for tumor treatment [8].

There are still some limitations to this study. Although we achieved the aim of partly relieving the tumor growth and slightly enhancing the therapeutic efficacy in TNBC treatment, the tumor growth was not completely inhibited or regressed. Here, we only performed a monotherapy PDT. As reported, the efficacy of combination therapy is better than a monotherapy [29–31]. Hence, it would be more effective to combine the biomimetic oxygen delivery PDT strategy with other therapies, such as chemotherapy, gene therapy, and immunotherapy. The therapeutic efficacy of these combination therapies still needs to be further validated.

## Conclusions

In summary, we successfully designed a biomimetic oxygen delivery nanoprobe CCm–HSA–ICG–PFTBA, in which the PFTBA core could dissolve a large amount of oxygen and the cancer cell membrane coating enabled homologous targeting and immune evasion abilities. We used a non-invasive and dynamic ^18^F-FMISO PET/CT imaging to measure hypoxia levels in vivo*,* and proved prominently hypoxia reduction at tumor sites. The therapeutic efficacy of PDT was further enhanced after the administration of the CCm–HSA–ICG–PFTBA because of the oxygen delivery, without causing notable additional side effects to the treated animals. Since the HSA, ICG, and PFTBA used in the nanoprobe are all FDA-approved and highly biocompatible, the nanoprobe may have the potential for clinical translation as an effective oxygen delivery agent to relief tumor hypoxia. Besides, there are many other therapies influenced by oxygen levels, such as radiotherapy [32], immunotherapy [33], chemotherapy [34], and sonodynamic therapy [35]. This strategy of biomimetic oxygen delivery nanoprobe could be a promising method to enhance the efficacy of hypoxia-limited therapies.

## Methods

### Materials

The HSA, ICG, and perfluorotributylamine (PFTBA, 98%) were purchased from Sigma-Aldrich (St. Louis MO, USA). RPMI-1640 medium, phosphate buffer saline (PBS), trypsin and ethylenediaminetetraacetic-acid (EDTA), penicillin–streptomycin, and fetal bovine serum (FBS) were purchased from Gibco Life Technologies (Gaithersburg MD, USA). 4′,6-diamidino-2-phenylindole (DAPI), paraformaldehyde and Cell Counting Kit-8 (CCK-8) were purchased from Boster Biotechnology (Wuhan, China). Hypoxyprobe plus kit were purchased from North Pacific International Inc. Singlet Oxygen Sensor Green (SOSG) was purchased from Thermo Fisher Scientific Inc. (Shanghai, China). Reactive Oxygen Species Assay Kit (DCFH-DA) were purchased from Beijing Solarbio Science & Technology Co., Ltd. (Beijing, China). ^18^F-FMISO NITTP were purchased from Huayi Isotopes Co. (Jiangsu, China). All of the aqueous solutions were prepared using deionized water (DI water) purified with a purification system. The other reagents used in this work were purchased from Aladdin-Reagent (Shanghai, China).

### Preparation of CCm–HSA–ICG–PFTBA

HSA (20 mg) was mixed in deionized water (1 mL) with stirring for 10 min. ICG dissolved in DI water (1 mg/mL) then dispersed in HSA solution and shake for 30 min at 37 ℃ to obtain HSA–ICG. The PFC (0.1 mL) was added gradually under sonication at 300 W in an ice bath for 8 min (ultrasonic for 7 s and rest for 3 s in every 10 s) to formulate HSA–ICG–PFTBA. Free ICG was removed by ultrafiltration centrifuge tube (Millipore molecular weight cutoff = 30 kDa).

Cancer cell membrane derivation could be achieved by emptying harvested 4T1 cells of their intracellular contents using a combination of hypotonic lysing, mechanical membrane disruption, and differential centrifugation according to the previous report [18]. The CCm coated on the surface of HSA–ICG–PFC were fabricated by the approach used in our previous study as reported [18]. HSA–ICG–PFC solution (1 mL) mixed with the prepared CCm–vesicles at different proportions. The mixture was subsequently extruded 11 times through 400 nm porous polycarbonate membrane. The resulting CCm–HSA–ICG–PFC were kept in PBS at 4 ℃ for further use.

### Characterization of CCm–HSA–ICG–PFC

The hydrodynamic diameter and zeta potential were measured by dynamic light scattering (DLS; Man 0486, Malvern, UK). The morphology and structure of HSA–ICG–PFC, CCm–HSA–ICG–PFC and CCm–vesicles will be characterized by transmission electron microscope (TEM; Talos F200X, FEI, Netherlands). The TEM samples were prepared by contacting the droplet containing HSA–ICG–PFC, CCm–HSA–ICG–PFC or CCm-vesicles with the copper grids for 60 s, negatively stained with 1% phosphotungstic acid for 30 s and dried with absorbent paper before the characterization. The stability experiments were carried out by measuring HSA–ICG–PFC and CCm–HSA–ICG–PFC in 1× PBS for 5 days using DLS for monitoring dynamic diameter.

The fluorescence of ICG was measured by the multifunctional microplate reader. The photoexcitation wavelength was 710 nm and the emission wavelength was 740–850 nm. The photostability of ICG was measured by 808 nm laser irradiation (1 W/cm^2^) to different samples (ICG, 2 μg/mL), and recording the absorption every 10 s for 1 min. The storage stability of ICG in different samples was performed by UV–vis spectra under dark condition till 60 h. The release of ICG in CCm–HSA–ICG–PFTBA and HSA–ICG–PFTBA (80 μg/mL) was determined by putting two samples into the dialysis bag (MWCO10k), and the dialysis bag was put into 15 mL of plasma, as release medium. The release of ICG in plasma was detected at 2, 4, 8, and 12 h by the UV–vis spectra and calculated based on the standard curve.

Oxygen release experiment was performed with a dissolved oxygen meter, to measure the oxygen concentrations in different solutions. Sample solutions (10 mL) were preoxygenated, and added into 50 mL deoxygenated water. The oxygen concentration in the water was monitored and recorded every 5 s for 800 s with a dissolved oxygen meter.

### In vitro ^1^O_2_ and ROS evaluation

SOSG was applied to detect the ^1^O_2_ generation of these samples. 100 μL different samples with the same concentration of ICG (50 μg/mL) and 20 μL SOSG (50 μM) were added into a black 96-well plate. With 808 nm laser irradiation, the fluorescence of oxidized SOSG (Ex/Em = 504/525 nm) was recorded every 10 s by multifunctional microplate reader.

DCFH-DA (Ex/Em = 495/529 nm) was used to indicate the ROS by confocal laser scanning microscope (CLSM). The 4T1 cells were seeded in confocal glass bottom dish with a density of 1 × 10^4^ cells. After incubated for 24 h, medium containing CCm–HSA–ICG–PFTBA, HSA–ICG–PFTBA, HSA–ICG and PBS were added to the dishes at the concentration of 10 μg/mL ICG for 3 h incubation. After washing for 3 times by PBS, the medium containing DCFH-DA (25 μM) was added to incubate with cells for 30 min. After washing for 3 times by PBS, cells were divided into two lines, with or without 808 nm laser irradiation (2 W/cm^2^) for 20 s (30 s pause after each 10 s irradiation). Then the cells were fixed by 4% polymer formaldehyde and the cell nucleus were labeled with 4′,6-diamidino-2-phenylindole (DAPI). CLSM was used to detect the green fluorescence of DCF.

Flow cytometry was applied to quantitatively reflect ROS generation. The procedure was similar to that for fluorescence imaging. The 4T1 cells were seeded in 6-well plates at the density of 1 × 10^5^ cells and stained by DCFH-DA (25 μM) for 30 min. After 808 nm laser irradiation (2 W/cm^2^) for 20 s (30 s pause after each 10 s irradiation), the cells were centrifuged, re-suspended in 300 mL PBS and analyzed by flow cytometry. The green fluorescence was detected on FL1 channel (Ex/Em = 488/525 nm).

### In vitro cytotoxicity

A CCK-8 assay was used to evaluate the enhanced PDT efficacy of CCm–HSA–ICG–PFTBA. 4T1 cells were seeded in 96-well plates at a density of 5 × 10^3^ cells per well and cultured for 12 h. CCm–HSA–ICG–PFTBA, HSA–ICG–PFTBA, and HSA–ICG were added to incubate with cells for 3 h at various concentrations of ICG (i.e., 1.25, 2.5, 5, 7.5, 10, 20, and 40 μg/mL). The saline group was used as control. Then the cells were irradiated by 808 nm laser (2 W/cm^2^) for 20 s (30 s pause after each 10 s irradiation). After 2 h co-incubation, cells were washed by PBS, and fresh culture medium was added. After further 24 h incubation, the fresh culture medium without serum (90 μL) mixed with CCK-8 (10 μL) was added into wells and the plates were incubated for another 2 h. Finally, the absorbance values of the cells per well were determined with a microplate reader (Bio-rad, Hercules CA, USA) at 450 nm for analyzing the cell viability. The background absorbance of the well plate was measured and subtracted.

### Animals and tumor models

Animals received care under the instruction of the Guidance Suggestions for the Care and Use of Laboratory Animals. Balb/c female mice (6 weeks) were purchased (Beijing HuaFuKang Bioscience Co. Ltd, China). To obtain tumor-bearing mice, hairs on the upper limb were removed. Then, 1 × 10^7^ 4T1 cells were subcutaneously injected into the right upper limb of each mouse. The tumor bearing mice was used for further experiments when the tumor volume reached 60–250 mm^3^.

### In vivo fluorescence imaging

When the volumes of tumor reached 100–150 mm^3^, the BALB/c mice were divided into four groups randomly. CCm–HSA–ICG–PFTBA, HSA–ICG–PFTBA, HSA–ICG (200 μL, 0.8 mg/kg for ICG), and saline were intravenously injected into tumor-bearing mice via the tail vein. All mice were anesthetized by isoflurane. The fluorescence images of mice at different time points (0, 3, 6, 12, 24, 36, and 48 h) were obtained by imaging system (Ex/Em = 710/790 nm). Then all mice were sacrificed to obtain the major organs (including heart, lung, liver, spleen, and kidney) and tumors to conduct the ex vivo fluorescence imaging.

### In vivo micro PET/CT imaging

PET/CT imaging was performed on a micro PET/CT (Trans-PET Discoverist 180, Raycan Technology Co., Ltd., Suzhou, China). ^18^F-FMISO and ^18^F-FDG were produced by PET Center, Union Hospital (Wuhan, China). For ^18^F-FMISO PET/CT imaging, on Day 1, each mouse was injected with 5.55 MBq (150 μCi) of ^18^F-FMISO via the tail vein. Then on Day 2, mice were divided into four groups and injected with CCm–HSA–ICG–PFTBA, HSA–ICG–PFTBA, HSA–ICG (200 μL, 0.8 mg/kg for ICG), and saline, respectively. 24 h later, on Day 3, mice were again injected with 5.55 MBq (150 μCi) of ^18^F-FMISO via the tail vein. Static scans of 10 min duration were acquired starting at 1 h post injection with ^18^F-FMISO, and the mice were maintained under isoflurane anesthesia during the scanning period.

For ^18^F-FDG PET imaging, mice in each group were randomly selected and injected with 5.55 MBq (150 μCi) of ^18^F-FDG via the tail vein. 10 min static scans were acquired at 1 h post injection. All the mice for ^18^F-FDG PET imaging were fasted overnight prior to the probe injection, maintained under isoflurane anesthesia and kept warm during the injection, waiting phase, and scanning periods.

The images were reconstructed using the orderedsubset expectation maximization (OSEM) algorithm. For each micro PET image, 3.0 mm diameter spherical regions of interest (ROIs) were drawn over the liver, tumor, and the contralateral muscle on the decay-corrected images using Amide to obtain the percentage of injected dose per gram-tissue (%ID/g) and measure the SUVmax of tumor, liver, and calculate the tumor to contralateral muscle (T/M) ratio. The highest uptake point of the entire tumor and liver was included in the ROI, and no necrosis area was included.

### Ex vivo immunofluorescence staining

Hypoxyprobe plus kit was used to stain tissues and detect hypoxia. Tumor-bearing mice were injected with CCm–HSA–ICG–PFTBA, HSA–ICG–PFTBA, and HSA–ICG (200 μL, 0.8 mg/kg for ICG) via tail vein, and divided into six groups (0, 6, 12, 24, 36, and 48 h). Then pimonidazole hydrochloride (60 mg/kg, Hypoxyprobe plus kit) was injected into the mice via tail vein. After 90 min later, all mice were sacrificed to obtain tumors for immunofluorescence staining following the protocols [36]. Hypoxia were stained with green fluorescence, cell nucleus were stained with DAPI and showed blue fluorescence, and blood vessels were stained with anti-CD31 and showed red fluorescence. All slices were examined by CLSM.

### In vivo photodynamic therapy and systematic toxicity

When tumor size reached about 60 mm^3^, the mice were randomly divided into eight groups (n = 6). The treatment groups were as follows: CCm–HSA–ICG–PFTBA (NIR), CCm–HSA–ICG–PFTBA, HSA–ICG–PFTBA (NIR), HSA–ICG–PFTBA, HSA–ICG (NIR), HSA–ICG, saline (NIR), and saline. On Day 0, all groups were injected with different samples (200 μL, 0.8 mg/kg for ICG) via tail veins, respectively. After 24 h later, namely on Day 1, all NIR groups were treated with 808 nm laser irradiation (2 W/cm^2^) for 2 min (1 min pause after each 30 s irradiation). ^18^F-FDG PET imaging and photograph taken were performed on Day 2, 7, and 14 to evaluate the tumor burden. The length and width of the tumor and mice body weight were recorded every 2 days over 14 days. The tumor volumes were calculated according to this formula: V = D × d^2^/2 (D is the longest diameter of tumor, and d is the shortest diameter of tumor). Relative tumor volume was calculated as V/V_0_ (V_0_ is the original tumor volume on Day 0). On Day 14, mice were sacrificed and tumors were weighted and photographed.

For evaluating systematic toxicity, on Day 14, all mice were euthanized and their blood and major organs (heart, lung, liver, spleen, and kidney) were collected for blood biochemistry test (red blood cells (RBC), hemoglobin (HGB), hematocrit (HCT), mean corpuscular volume (MCV), mean corpuscular hemoglobin (MCH), mean corpuscular hemoglobin concentration (MCHC), white blood cells (WBC), lymphocytes percentage (Lymph%), monocyte percentage (Mon%), neutrophil percentage (Neu%), and platelets (PLT)), hematology tests [alkaline phosphatase (ALP), aspartate aminotransferase (AST), alanine transaminase (ALT), creatinine (CRE) and blood urea nitrogen (BUN)], and histology analysis (hematoxylin and eosin (H&E)-stained slices).

### Statistical analysis

Results are expressed as mean ± standard error of the mean. Data analyses were conducted using the software GraphPad Prism 6.0 (GraphPad Software, San Diego CA, USA). The differences among groups were analyzed using one-way ANOVA analysis followed by Tukey’s post-test. *P* value of < 0.05 indicates statistical significance.

## Supplementary Information

**Additional file 1:** **Fig. S1.** Stabilities in dark conditions. The absorbance of ICG in **a** CCm–HSA–ICG–PFTBA, **b** HSA–ICG–PFTBA, **c** HSA–ICG, and **d** ICG. **e** Normalized absorption of CCm–HSA–ICG–PFTBA, HSA–ICG–PFTBA, HSA–ICG, and ICG stored in dark till 60 h. **Fig. S2.** **a** In vivo coronal ^18^F-FMISO PET/CT images of TNBC xenografts before and after 24 h injection of the CCm–HSA–ICG–PFTBA, HSA–ICG–PFTBA, HSA–ICG, and saline. White arrows indicated tumor sites. Red arrows and L indicated livers. **b** The quantitative analysis of liver SUVmax of CCm–HSA–ICG–PFTBA, HSA–ICG–PFTBA, HSA–ICG, and saline groups in the pre and post ^18^F-FMISO PET/CT imaging. **Fig. S3.** Immunofluorescence images of tumor slices stained by the hypoxyprobe. The blood vessels and hypoxia areas were stained with anti-CD31 antibody (red) and antipimonidazole antibody (green), respectively. Scale bars = 100 μm. **Fig. S4.** ^18^F-FDG PET imaging. 4T1 xenograft mice were treated with CCm–HSA–ICG–PFTBA, HSA–ICG–PFTBA, HSA–ICG, and saline with or without NIR laser irradiation. ^18^F-FDG PET imaging was performed at **a** day 2, **b** day 7, and **c** day 14 after treatment (white arrows point to the tumors).

## Data Availability

All data generated or analyzed during this study are included in this manuscript.

## Abbreviations

TNBC: Triple-negative breast cancer
ER: Estrogen receptor
PR: Progesterone receptor
HER2: Human epidermal growth factor receptor 2
PDT: Photodynamic therapy
ROS: Reactive oxygen species
^1^O_2_: Singlet oxygen
PFC: Perfluorocarbons
PFTBA: Perfluorotributylamine
NIR: Near-infrared
ICG: Indocyanine green
FDA: U.S. Food and Drug Administration
HSA: Human serum albumin
CCm: Cancer cell membranes
CCm–HSA–ICG–PFTBA: Cancer cell membrane-coated human serum albumin–Indocyanine green–doped perfluorotributylamine
^18^F-FMISO: ^18^F-Fluoromisonidazole
PET/CT: Positron emission tomography/computed tomography
DLS: Dynamic light scattering
PBS: Phosphate-buffered saline
TEM: Transmission electron microscopy
SOSG: Singlet Oxygen Sensor Green
DCFH-DA: Dichloro-dihydro-fluorescein diacetate
H_2_DCF: 2,7-Dichlorofluorescin
CCK-8: Cell counting kit-8
RES: Reticuloendothelial system
T/M ratios: Tumor-to-muscle ratios
H&E: Hematoxylin and eosin
EPR effect: Enhanced permeability and retention effect
