# Supplementary material for: Biomimetic oxygen delivery nanoparticles for enhancing photodynamic therapy in triple-negative breast cancer
Source: J Nanobiotechnology. 2021 Mar 20;19:81. doi: 10.1186/s12951-021-00827-2 (PMC7981819; doi:10.1186/s12951-021-00827-2)
Supplement: Supplementary file 1 — Additional file 1: Fig. S1. Stabilities in dark conditions. The absorbance of ICG in a CCm–HSA–ICG–PFTBA, b HSA–ICG–PFTBA, c HSA–ICG, and d ICG. e Normalized absorption of CCm–HSA–ICG–PFTBA, HSA–ICG–PFTBA, HSA–ICG, and ICG stored in dark till 60 h. Fig. S2. a In vivo coronal 18F-FMISO PET/CT images of TNBC xenografts before and after 24 h injection of the CCm–HSA–ICG–PFTBA, HSA–ICG–PFTBA, HSA–ICG, and saline. White arrows indicated tumor sites. Red arrows and L indicated livers. b The quantitative analysis of liver SUVmax of CCm–HSA–ICG–PFTBA, HSA–ICG–PFTBA, HSA–ICG, and saline groups in the pre and post 18F-FMISO PET/CT imaging. Fig. S3. Immunofluorescence images of tumor slices stained by the hypoxyprobe. The blood vessels and hypoxia areas were stained with anti-CD31 antibody (red) and antipimonidazole antibody (green), respectively. Scale bars = 100 μm. Fig. S4. 18F-FDG PET imaging. 4T1 xenograft mice were treated with CCm–HSA–ICG–PFTBA, HSA–ICG–PFTBA, HSA–ICG, and saline with or without NIR laser irradiation. 18F-FDG PET imaging was performed at a day 2, b day 7, and c day 14 after treatment (white arrows point to the tumors). [file 12951_2021_827_MOESM1_ESM.docx]

Biomimetic Oxygen Delivery Nanoparticles for Enhancing Photodynamic Therapy in Triple-negative Breast Cancer

Hanyi Fang^1,2^, Yongkang Gai^1,2^, Sheng Wang^3^, Qingyao Liu^1,2^, Xiao Zhang^1,2^, Min Ye^1,2^, Jianling Tan^1,2^, Yu Long^1,2^, Kuanyin Wang^1,2^, Yongxue Zhang^1,2^, Xiaoli Lan^1,2,^*

^1^ Department of Nuclear Medicine, Union Hospital, Tongji Medical College, Huazhong University of Science and Technology, Wuhan, 430022, China

^2^ Hubei Province Key Laboratory of Molecular Imaging, Wuhan, 430022, China

^3^ School of Pharmacy, Tongji Medical College, Huazhong University of Science and Technology, Wuhan, 430030, China

* Corresponding Author: Xiaoli Lan. E-mail: xiaoli_lan@hust.edu.cn


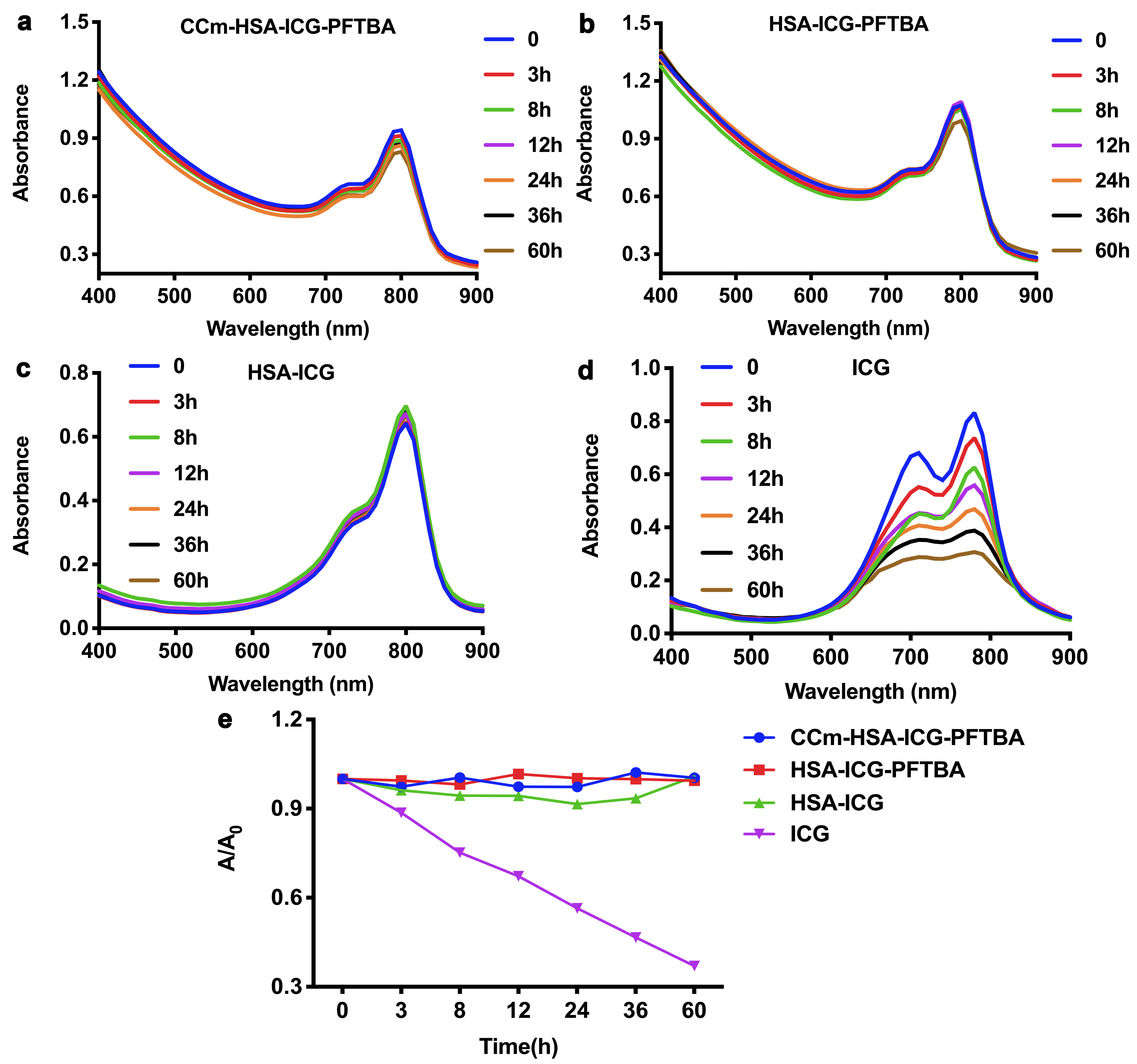


**Fig. S1** Stabilities in dark conditions. The absorbance of ICG in **a** CCm-HSA-ICG-PFTBA, **b** HSA-ICG-PFTBA, **c** HSA-ICG, and **d** ICG. **e** Normalized absorption of CCm-HSA-ICG-PFTBA, HSA-ICG-PFTBA, HSA-ICG, and ICG stored in dark till 60 h.


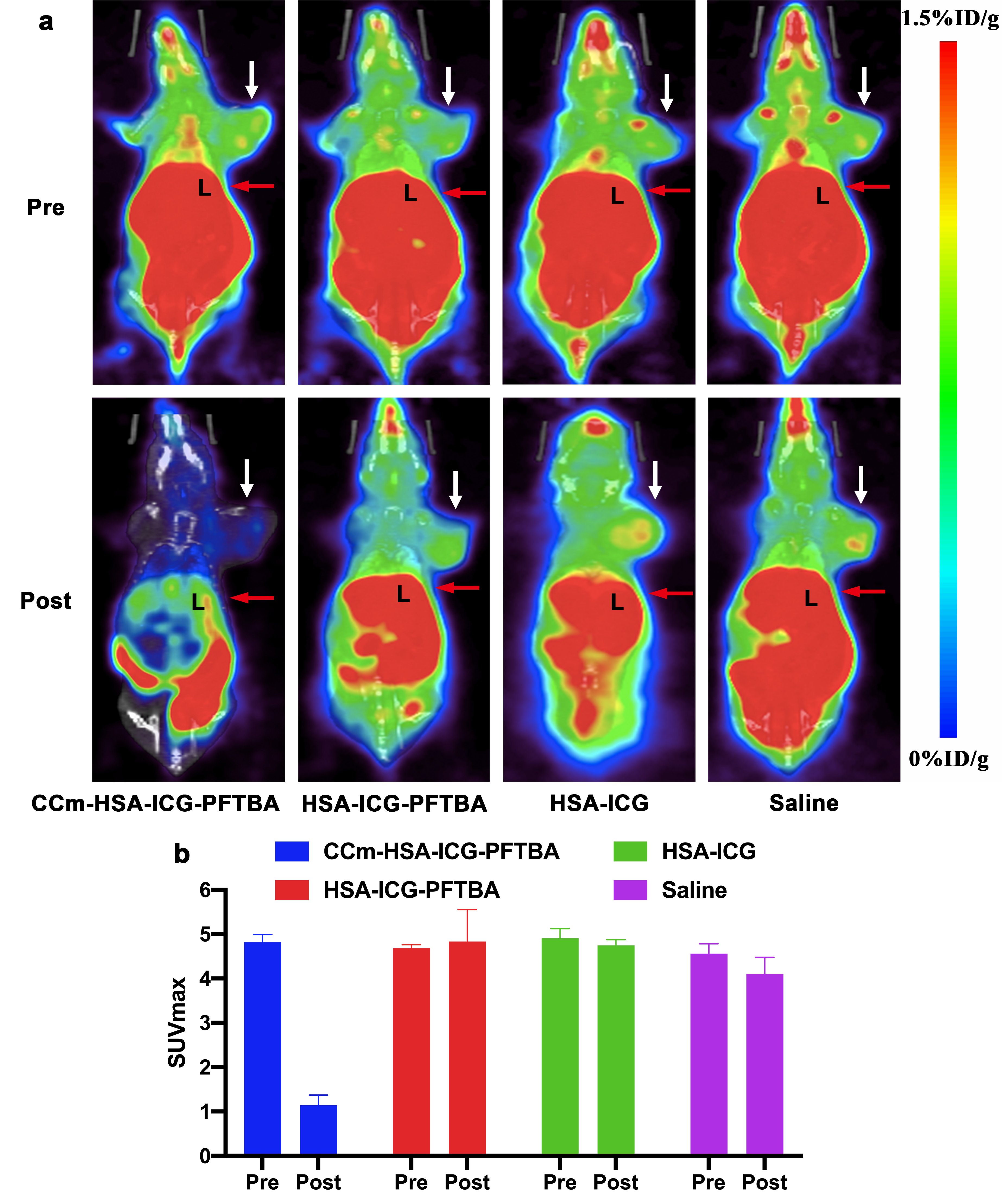


**Fig. S2** **a** *In vivo* coronal ^18^F-FMISO PET/CT images of TNBC xenografts before and after 24 h injection of the CCm-HSA-ICG-PFTBA, HSA-ICG-PFTBA, HSA-ICG, and saline. White arrows indicated tumor sites. Red arrows and L indicated livers. **b** The quantitative analysis of liver SUVmax of CCm-HSA-ICG-PFTBA, HSA-ICG-PFTBA, HSA-ICG, and saline groups in the pre and post ^18^F-FMISO PET/CT imaging.


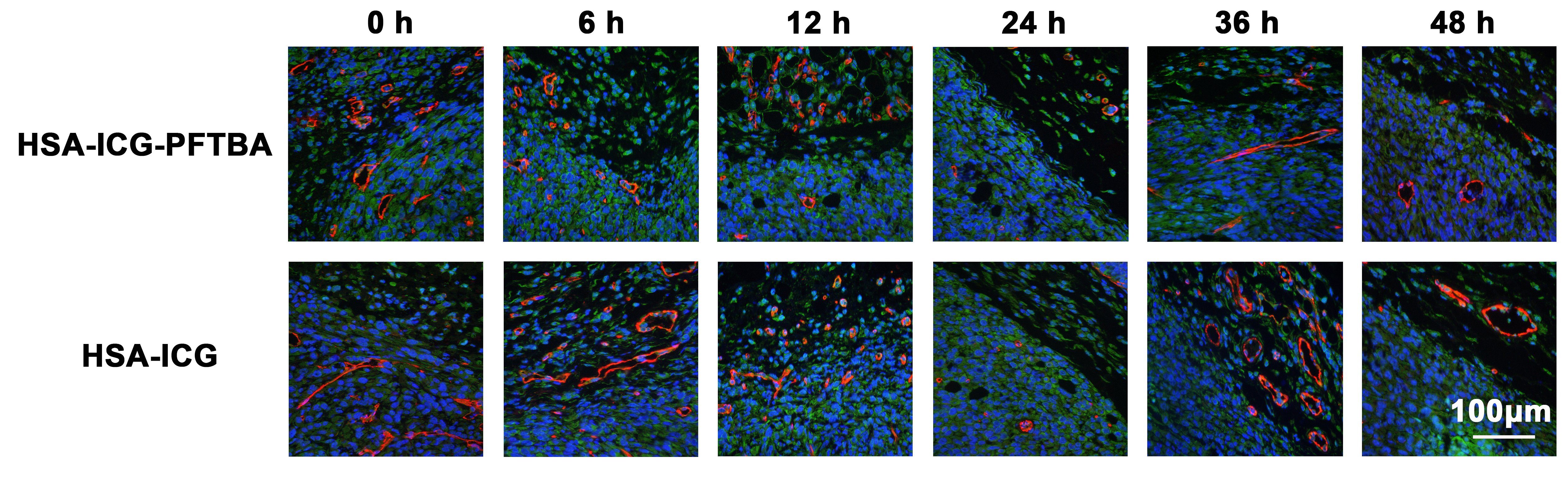


**Fig. S3** Immunofluorescence images of tumor slices stained by the hypoxyprobe. The blood vessels and hypoxia areas were stained with anti-CD31 antibody (red) and antipimonidazole antibody (green), respectively. Scale bars = 100 μm.


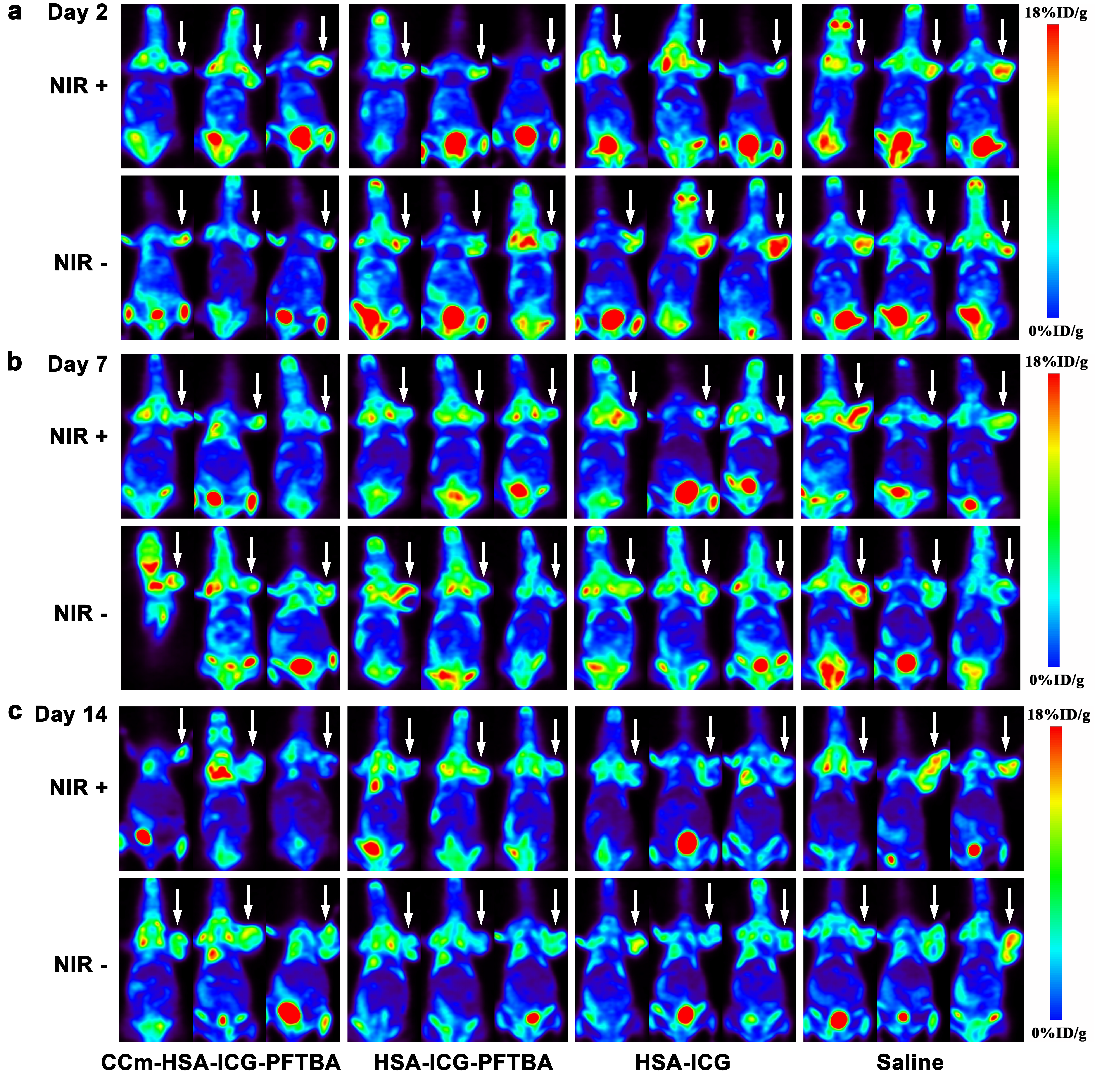


**Fig. S4** ^18^F-FDG PET imaging. 4T1 xenograft mice were treated with CCm-HSA-ICG-PFTBA, HSA-ICG-PFTBA, HSA-ICG, and saline with or without NIR laser irradiation. ^18^F-FDG PET imaging was performed at **a** day 2, **b** day 7, and **c** day 14 after treatment (white arrows point to the tumors)
